# T lymphocyte membrane bionic nanomedicine synergizes with radiotherapy for enhancing Mg^2+^-mediated tumor metallo-immunotherapy and preventing recurrence

**DOI:** 10.7150/thno.127717

**Published:** 2026-01-08

**Authors:** Yongjian Zhang, Shijian Liu, Xu Tong, Qi Li, Qianqian Gan, Shipeng Ning, Meiyin Zhang

**Affiliations:** 1 The Sixth Affiliated Hospital of Harbin Medical University, Harbin, Heilongjiang, 150000, China.; 2 Department of Surgical Oncology, Harbin Medical University Cancer Hospital, Harbin, 150081, China.; 3 Department of Nephrology, The Second Affiliated Hospital of Harbin Medical University, Harbin, 150001, China.; 4 Department of Breast Surgery, The Second Affiliated Hospital of Guangxi Medical University, Nanning, 530000 China.; 5 Research Center of Nanomedicine Technology, The Second Affiliated Hospital of Guangxi Medical University, Nanning, 530000, China.

**Keywords:** metallo-immunotherapy, T lymphocyte membranes, MgCO_3_ nanosheets, sustained ROS generation, tumor recurrence

## Abstract

**Background and Aim:** Magnesium ion (Mg²⁺)-mediated metallo-immunotherapy effectively promotes the activation of memory T cells, thereby helping to mitigate tumor recurrence following traditional treatments such as radiotherapy (RT). However, factors such as the acidity of the tumor microenvironment, along with the upregulated expression of immune checkpoints induced by RT and Mg²⁺, may compromise its therapeutic efficacy.

**Material and Methods:** In this work, we developed a T cell membrane-coated, hemin-loaded magnesium carbonate nanomedicine (designated as THM). Following intravenous injection, THM catalyzes the hydrogen peroxide generated during RT to induce a burst of reactive oxygen species (ROS), thereby producing a tumor vaccine that promotes dendritic cell maturation and T cell activation. Simultaneously, THM reacts with H⁺ to mitigate the acidic tumor microenvironment while releasing Mg²⁺, which further enhances the generation and activation of central memory T cells (Tcm) to confer long-term anti-tumor immunity following RT.

**Results:** RT combined with Mg²⁺ treatment upregulates PD-L1 expression in tumor cells. Notably, the PD-1 protein on THM can competitively bind to PD-L1, thereby mitigating the side effects associated with the combined therapy. *In vitro* and *in vivo* data confirm that this combinatorial therapy boosts Tcm-mediated antitumor activity, mitigates treatment-induced immune suppression, and potently prevents tumor recurrence.

**Conclusions:** This work provides critical insights for the clinical translation of antitumor immunotherapy.

## Introduction

Cancer remains an urgent challenge 1-4. Magnesium serves as a cofactor for many intracellular enzymes and participates in key processes such as DNA synthesis, repair, and cell cycle regulation 5-7. Magnesium ions play crucial roles in maintaining cell membrane stability, regulating intracellular signal transduction, and contributing to energy metabolism 8. Research indicates that magnesium ions can influence cell proliferation, differentiation, and apoptosis, thereby participating in the initiation and progression of tumors 9, 10. Magnesium can be sensed by T-cell lymphocyte function-associated antigen-1 (LFA-1) 9. The concentration of magnesium ions (Mg^2+^) can regulate the conformation of LFA-1, promoting T-cell activation. Although promising, magnesium ion-mediated metal immunotherapy can promote PD-1 expression in T cells, which in turn inhibits T-cell function 9. Developing combination therapies represents an effective approach to enhance magnesium ion-mediated Metallo-Immunotherapy. Although there are currently some magnesium-based nanomaterials used in tumor immunotherapy, the role of magnesium ions in the regulation of immunotherapy has been overlooked 11, 12. Furthermore, how to precisely deliver magnesium ions to tumor tissues needs to be addressed.

In recent years, nanomedicine has attracted extensive attention in the field of cancer therapy 4, 13-17. Cell membrane-mimicking nanomedicines offer new ideas for magnesium ion-mediated Metallo-Immunotherapy. Cell membrane biomimetic nanotechnology, by mimicking the composition and function of cell membranes, can effectively improve the biocompatibility of tumor-targeted drug delivery systems 18. This technology utilizes the abundant membrane proteins on cell membranes to achieve precise recognition of tumor cells. Certain cell membrane biomimetic nanoparticles demonstrate nearly 80% increased affinity for tumor cells, significantly enhancing the targeting capability of drug delivery 18-24. PD-1 high-expressing T cell membranes (TCM) represent a unique type of cell membrane capable of targeting tumor tissues 25, 26. Unlike other cell membranes, TCM not only target tumor cells but also competitively block PD-L1-mediated T cell exhaustion. Given these properties, we propose TCM-biomimetic MgCO_3_ for targeted Mg²⁺ delivery and concurrent PD-L1 receptor blocking to mitigate Mg²⁺-induced side effects. Furthermore, magnesium carbonate nanoparticles (MgCO_3_) can consume hydrogen ions to alleviate the inhibitory effect of acidic microenvironments on T cells.

Relying solely on the anti-tumor effect mediated by magnesium ions may not effectively activate anti-tumor immunity. We plan to combine magnesium ion therapy with radiotherapy (RT). RT elicits its anti-tumor immune effects primarily by inducing immunogenic cell death (ICD) in tumor cells 27-31. Dendritic cells (DCs) and macrophages detect these danger signals through their pattern recognition receptors, which triggers DC maturation 32, 33. This process ultimately enhances tumor-specific T cell immunity by facilitating antigen presentation and co-stimulation 34-36. Thus, combining RT with magnesium ion-mediated Metallo-Immunotherapy may induce a potent anti-tumor immune response.

In this work, we designed a T cell membrane-coated, Hemin loaded magnesium carbonate nanomedicine (named THM). Hemin (hemin chloride) is the natural form of iron porphyrin compounds. As the core structure of the active center of hemoglobin, myoglobin, and peroxidases (e.g., horseradish peroxidase, HRP), it inherently possesses endogenous peroxidase-mimetic activity (POD-like activity) 37. In recent years, Hemin-based tumor catalytic therapy mediated by its POD enzymatic activity has emerged as a research hotspot 38, 39. Post intravenous injection, THM catalyzes RT-derived H₂O₂ to induce ROS burst, triggering tumor cell ICD and promoting dendritic cell maturation and T cell activation. Simultaneously, THM reacts with H^+^ to alleviate the acidic microenvironment while releasing Mg^2+^ that further enhance central memory T cell (T_CM_) production and activation for long-term anti-tumor immunity. Notably, RT and Mg²⁺ treatment enhance PD-L1 expression in tumor cells. However, the PD-1 protein on THM can competitively bind to PD-L1 protein, mitigating the side effects of the combined therapy. Overall, this integrative therapeutic approach potentiates the tumor-fighting properties of T_CM_, mitigates immunosuppressive effects associated with combination therapies, and suppresses tumor relapses. These findings hold substantial promise for informing future clinical strategies targeting anti-tumor immunity.

## Materials and Methods

### Materials

Magnesium carbonate (MgCO_3_) nanosheets were purchased from Yumu (Ningbo) New Materials Co., LTD. The other reagents used in this work were purchased from Sinopharm Chemical Reagent (China) and Shanghai Macklin Biochemical Technology Co., Ltd. (China).

### Preparation and characterization of T cell membrane (TCM)-coated MgCO_3_ (TM), and hemin-loaded TM (THM)

Based on the previous method, the TCM were obtained from EL4 cells 25. Then, aqueous solution of MO (0.2 mg/mL, 1 mL) and 1 mg TCM and then repeatedly coextruded through 200 nm pores by using a physical extruder (‌Avestin). The resultant TMO particles were centrifuged and washed with PBS several times to remove the excess TM. PMO and RMO were prepared in the same way as TMO.

Protein expression was determined by western blot. The zeta potentials were measured by DLS. The morphology of synthesized materials was observed with field-emission TEM (JEM-F200). The thickness of nanomaterials was conducted using an Atomic Force Microscope (Dimension Edge, Bruker).

### Mg²⁺ release study

*In vitro* Mg²⁺ release from THM (10 mL, containing 20 μg hemin) was assessed in PBS at pH 7.4 or 6.0 to evaluate H⁺ stimuli effects. At designated time points, 100 μL aliquots were collected, and released Mg²⁺ levels were quantified via ICP-AES.

### ROS detection

Hydroxyl radical generation in THM was detected by TMB colorimetric reaction: THM (hemin 5 μg/mL final conc.) was mixed with TMB (5 μg/mL final conc.) in pH 7.4 PBS, supplemented with 10 mM H₂O₂, and TMB absorbance was measured every 3 min via UV-vis spectrophotometer.

### POD-like activity of THM

To examine the enzyme-like activity of the THM, catalytic oxidation experiments were performed according to previous reports with modifications 40. The maximum reaction velocity (V*_max_*) and Michaelis constant (K*_m_*) of obtained nanozymes were calculated following the Michaelis-Menten equation:



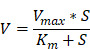



*V_max_* represented the maximal reaction velocity, S was the substrate concentration, and K*_m_* was the Michaelis constant reflecting the affinity of the nanozymes towards the substrate.

### Cell culture

4T1 mouse breast cancer cell line was obtained from the Cell Bank of the Chinese Academy of Sciences and incubated in RPMI-1640 medium supplemented with 10% FBS in a humidified atmosphere at 37 ℃.

*In vitro* cell experiments include transwell experiment, DNA double-strand breaks and clonogenic survival assay were conducted according to the previous work 34, 41, 42.

### *In vitro* cancer targeting assay

4T1 cells seeded in 24-well plates were cultured for 12 h. 100 μL Dil-labeled RHM, THM (10 μg/mL hemin) or anti-PD-L1-contained THM was added, and cells were incubated for 1 h. After Lyso-Tracker Green staining, cells were fixed, stained with DAPI, and imaged by CLSM.

### Tumor spheroid penetration test

For the permeability assay, 4T1 multicellular tumor spheroids were cultured in ultra-low adsorption plates (QINGDAO AMA) for 3 days until reaching ~150 μm in diameter. The spheroids were then incubated for 12 h in medium containing DiO-labeled THM or RHM. CLSM was used to detect the green fluorescence of DiO (Ex: 495 nm, Em: 519 nm) across different spheroid sections.

### Intracellular ROS and immunogenic cell death (ICD) detection

4T1 cells (1.5 × 10⁵ cells/well) were seeded in plates for 12 h and allocated to 5 groups: PBS, RT (4 Gy), THM, RHM+RT, THM+RT (MgCO₃: 0.1 mg/mL). DCFH-DA was used at different time points post-RT according to the instructions. Cells were washed and incubated with anti-Calreticulin/HMGB1 antibody (Bioss) and fluorescent secondary antibody (30 min), stained with DAPI (20 min), and observed under CLSM after washing. Cell medium was analyzed via ATP Assay Kit (Beyotime) and ELISA.

### Animal tumor models

Female Balb/c mice aged 5-6 week were purchased from Vital River Company (Beijing, China). All animal procedures were performed in accordance with the guidelines for Care and Use of Laboratory Animals of the Ministry of Health in People's Republic of PR China and approved by the Animal Ethics Committee of Guangxi Medical University (Approval number: 2025-KYL (017)).

### Western blot and T cells response measurements experiments

CD8^+^ T cells from spleens were activated by Ova peptide (0.5 μM) in the presence of 5 groups for 48h: (1) PBS; (2) Hemin; (3) HM; (4) THM and (5) THM+BIRT377 (10 μM). Each group of materials was pre-soaked in PBS solution with a pH of 6.0. MgCO_3_ concentration was 0.1 mg/mL, followed by stimulation for 2 days. The expression of PGC1α and β-Actin was analyzed by western blot. The secretion of IL-2 in the supernatant and expression of MitoFM, mitochondrial ATP, CD69, IFN-γ and Granzyme B (GZMB) in CD8^+^ T cells was detected by ELISA and flow cytometry. The upper layer of the medium was added to the plates pre-seeded with 4T1-OVA tumor cells (3×10^4^ per well in 24-well plates), and the concentration of LDH in the supernatant were detected by LDH Assay Kit.

### *In vivo* pharmacokinetics and biodistribution study

Balb/c mice received an intravenous injection of 100 μL PBS containing MgCO_3_ or THM (with an equivalent MgCO_3_ dose of 20 mg/kg). At various time points after the injection, 20 μL blood plasma was collected and Mg^2+^ concentration was quantitatively analyzed by ICP-MS. To study the biodistribution of THM in various organs, Balb/c mice bearing 4T1 tumor (n = 3) received treatment as mentioned above. The main organs were stripped at predetermined time points and digested and measured by ICP-MS.

### *In vivo* anti-tumor study

Balb/c mice were subcutaneously injected with 5 × 10^6^ 4T1 cells into the right flank. When the tumor grows to approximately 100 mm^3^, treatment is carried out on day 0. The mice were firstly divided into different groups (Each group included 5 mice): (1) PBS; (2) RT (4 Gy); (3) THM; (4) RHM+RT and (5) THM+RT. The MgCO_3_ dose was 40 mg/kg. Mice body weight and tumor volume in all groups were monitored every 6 days. A caliper was employed to measure the tumor length and tumor width, and the tumor volume was calculated according to following formula. Tumor volume = tumor length × tumor width^2^ / 2. Tumor size, weights and survival curves were continuously measured for 60 days. Another group of mice underwent the same experiment and were sacrificed after 15 days. Five main organs and tumors were weighed and subjected to tissue section analysis. Immune cell analysis and inflammatory factor detection were conducted on different tissues and organs.

### Statistical analysis

For variance analysis, One-way analysis of variance (ANOVA) with Tukey's post hoc test was used. p values of <0.05 were considered significant. *p < 0.05, **p < 0.01, ***p < 0.001.

## Results and Discussion

### Preparation and characterization of THM

**Figure 1:**
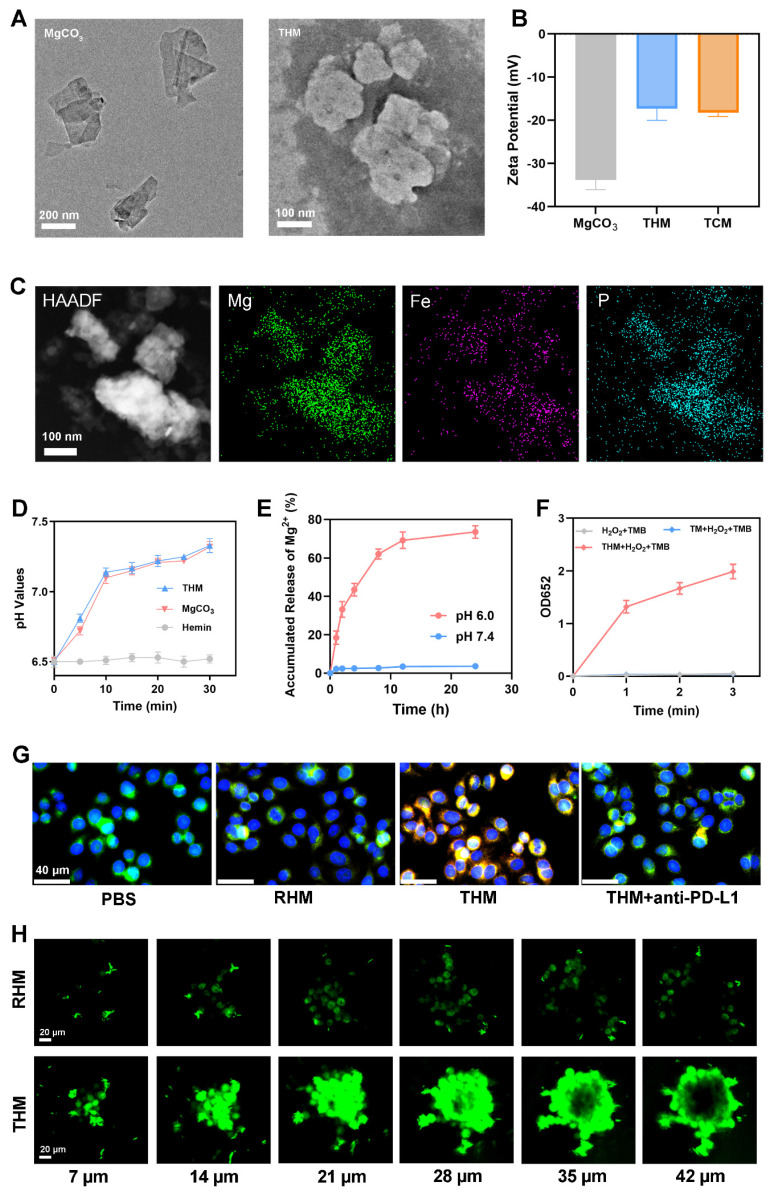
(A) TEM image of MgCO_3_ and THM. (B) Zeta potential of indicated formulations. (C) HAADF-SETM images and element mapping of THM. (D) pH values of PBS after incubation with different formulations. (E) Mg^2+^ release profile. (F) The absorption value at 652 nm (oxTMB) in different groups (n = 3). (G) CLSM images of cancer cells incubated with Dil labeled RHM (Red blood cell membrane-coated MgCO_3_/Hemin nanoparticles), THM or THM (containing anti-PD-L1 antibody) for 1 h (Blue: DAPI; green: Lyso-tracker green; red: Dil). (H) Penetration of DiO labeled nanoparticles in multicellular tumor spheroids by z-stack scanning of CLSM**.**

As shown in **Figure 1A**, the magnesium carbonate nanoparticles were lamellar. The THM surface shows obvious protein negative staining membranes structure. The atomic force microscopy (AFM) images were shown in **Figure S1**. Western blot showed high PD-1 expression in both TCM and THM (**Figure S2**). The zeta potentials of TCM and THM exhibit similarities (**Figure 1B**), which further confirms the presence of the cell membrane in THM. THM has good stability (**Figure S3**). High-angle annular dark-field scanning TEM (HAADF-STEM) images and corresponding elemental maps taken on THM further confirmed the successful Hemin loading and TCM coating. This is evidenced by the presence of iron and phosphorus elements (**Figure 1C**). The drug loading of Hemin was 24.1±2.5% by HPLC. As a control, TCM-coated MgCO_3_ (TM) were generated. The pH increased following the addition of MgCO₃ and THM (**Figure 1D**).** Figure 1E** confirms acid-responsive Mg²⁺ release from THM. THM then catalyzed TMB oxidation to oxTMB, causing an absorbance shift at 652 nm (**Figure 1F and S4**). The obtained Michaelis-Menten parameters (**Figures S5**) indicate a *K_m_* value of 1.54 mM and a *V_max_* of 9.57 × 10⁻^9^ M/s. To evaluate the tumor-targeting capability of THM, red blood cell membrane-coated MgCO_3_/Hemin (RHM) was prepared as a control. Because there is no specific protein targeting tumor cells on the surface of red blood cell, and it has a phospholipid structure similar to THM 24, 43, 44, it can be used as a control group of THM. Confocal laser scanning microscopy (CLSM) results exhibited good tumor targeting of THM (**Figure 1G**). CLSM z-stack scanning revealed that RHM fluorescence was confined to tumor spheroid edges, while THM achieved efficient deep tumor penetration compared with the RHM group (**Figure 1H**), thereby verifying THM's capacity to improve delivery depth in dense tumor microenvironments.

### *In vitro* anti-tumor ability of THM

**Figure 2:**
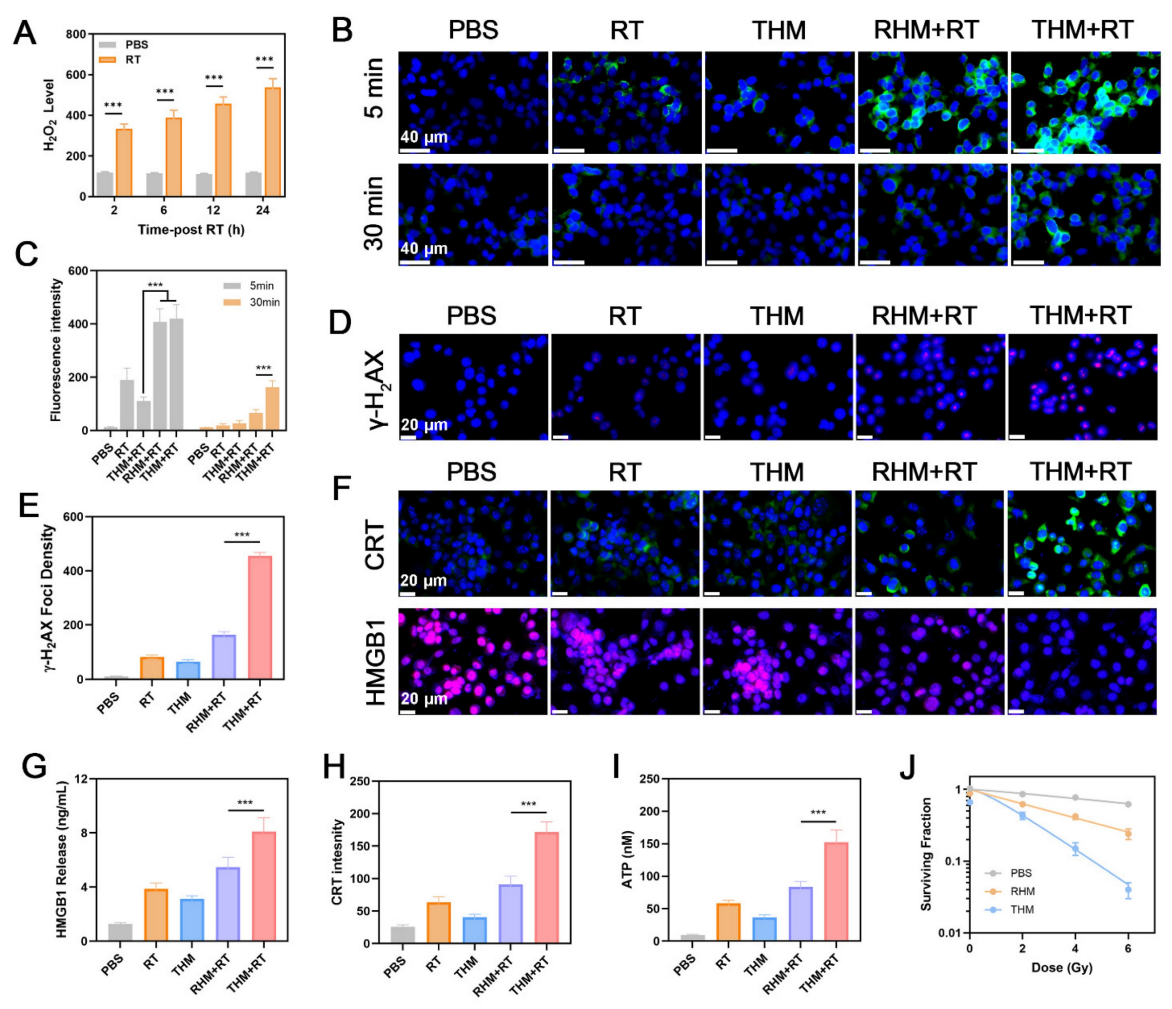
(A) Production of intracellular H_2_O_2_ by RT. (B) CLSM images of total ROS in 4T1 cells. RT: 4Gy. (C) ROS fluorescence intensity of Figure 2B. (D) Expression of γ-H_2_AX (red fluorescence) and (E) γ-H_2_AX Foci density from 4T1 cells. RT: 4Gy. (F) Expression of HMGB1 and CRT and (G) HMGB1 release from 4T1 cells. (H) CRT fluorescence intensity of Figure 2F. (I) ATP release from 4T1 cells. (J) Clonogenic survival assay (n=3). ^***^p < 0.001.

Previous studies have shown that radiotherapy can stimulate tumor cells to produce hydrogen peroxide (H_2_O_2_) 28. To verify this, we first detected H_2_O_2_ production in tumor cells after radiotherapy (**Figure 2A**). Relative to the blank control group, RT induced marked time-dependent H₂O₂ production. CLSM further detected intracellular ROS generation across treatments. As shown in **Figures 2B and 2C**, within 5 minutes of treatment, compared with control groups, radiotherapy significantly induced ROS production. Notably, even 30 minutes after treatment, the THM+RT group still exhibited strong ROS fluorescence signals. This enhanced ROS generation is attributed to Hemin's POD-like activity, which makes ROS production more persistent. Furthermore, we confirmed through cell experiments that THM generates hydroxyl radicals within cells, rather than other types of ROS (**Figure S6**).

Radiotherapy can directly cause DNA breaks by acting on organic molecules, which can be reflected by γ-H_2_AX detection 45-47. As shown in **Figures 2D and 2E**, researchers examined DNA damage in 4T1 cells to verify the radiosensitization effect of THM nanoparticles. Compared with the control group, cells treated with THM combined with radiotherapy showed a large amount of red fluorescence, confirming that THM+RT can lead to extensive DNA damage. Previous studies have shown that effective ROS generation can trigger ICD 48. Therefore, we investigated the ability of the THM+RT group to induce ICD in vitro. Calreticulin (CRT) is a pro-apoptotic protein that can be transferred to the cell membrane surface during ICD as a "eat me" signal to stimulate anti-tumor immune responses 19, 44, 49. Immunofluorescence imaging showed that the THM+RT group significantly promoted the exposure of CRT on the surface of cancer cells compared with other control groups (**Figures 2F, 2H and S7**). Meanwhile, the THM+RT group showed markedly HMGB1 and ATP release (**Figures 2G and 2I**). As shown in **Figure 2J**, THM most effectively enhanced effect of RT, and this radiosensitization effect was dose-dependent on X-ray irradiation. These findings suggest that THM+RT can effectively induce ICD in tumor cells, which is crucial for enhancing tumor antigen presentation and T lymphocyte activation.

The activation of APCs serves as a pivotal step in adaptive anti-tumor immune responses 19, 40, 50. Among APCs, dendritic cells (DCs) play a central role in immunotherapy 51. During ICD, tumor cells release DAMPs 52. To evaluate the BMDCs maturation, we quantified the expression of the key co-stimulatory molecules CD80 and CD86.

**Figure 3:**
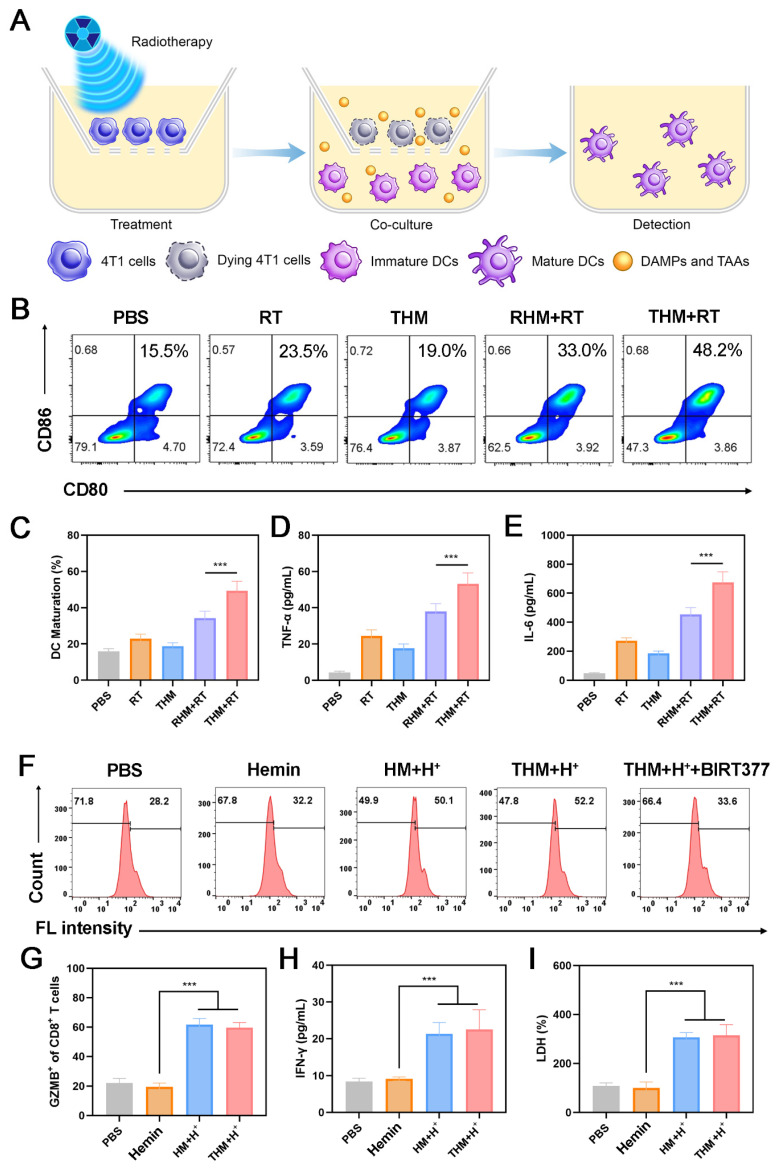
(A) Schematic illustration of maturation of BMDCs *in vitro*. (B) Flow cytometry analysis and (C) Quantitative analysis of mature BMDCs after different treatments. (D) The levels of TNF-α and (E) IFN-γ are secreted by mature BMDCs. (F) Flow fluorescence analysis of CD69 expression in CD8^+^ T cells. HM: Hemin+MgCO_3_. (G) Quantification of GZMB and (H) IFN-γ in CD8^+^ T cells. (I) Measurement of LDH levels (n = 3). ^***^p < 0.001.

As illustrated in** Figures 3A-3C**, the percentage of matured BMDCs in the THM+RT group was 1.46 times higher than RHM+RT group. Moreover, treatment with THM+RT elicited the tumor necrosis factor-α (TNF-α) and interleukin-6 (IL-6), as evidenced in **Figures 3D and 3E**. Collectively, these results verify that tumor cells treated with the THM+RT regimen are highly efficient in driving the maturation of BMDCs. CD8^+^ T cells were stimulated with Ova peptide under five distinct treatment conditions. Flow cytometry was subsequently utilized to assess the expression of CD69. As illustrated in **Figure 3F**, compared with control groups, hydrogen ion treatment of HM and THM could effectively promote the expression of CD69 on CD8^+^ T cells, thereby activating T cells. To further assess the effect of THM stimulation on T cell-mediated tumor cytotoxicity, we analyzed granzyme B (GZMB)^+^CD8^+^ T cells. Flow cytometry revealed a marked upregulation of GZMB in CD8^+^ T cells following THM treatment (**Figure 3G**). Additionally, THM-treated T cells showed a significant increase in IFN-γ secretion (**Figure 3H**). THM group exhibited the highest cytotoxic against cancer cells (**Figure 3I**). These findings collectively demonstrate that THM treatment robustly enhances antigen-specific T cell activation and tumor-killing efficacy.

### *In vivo* anti-tumor ability of THM

**Figure 4:**
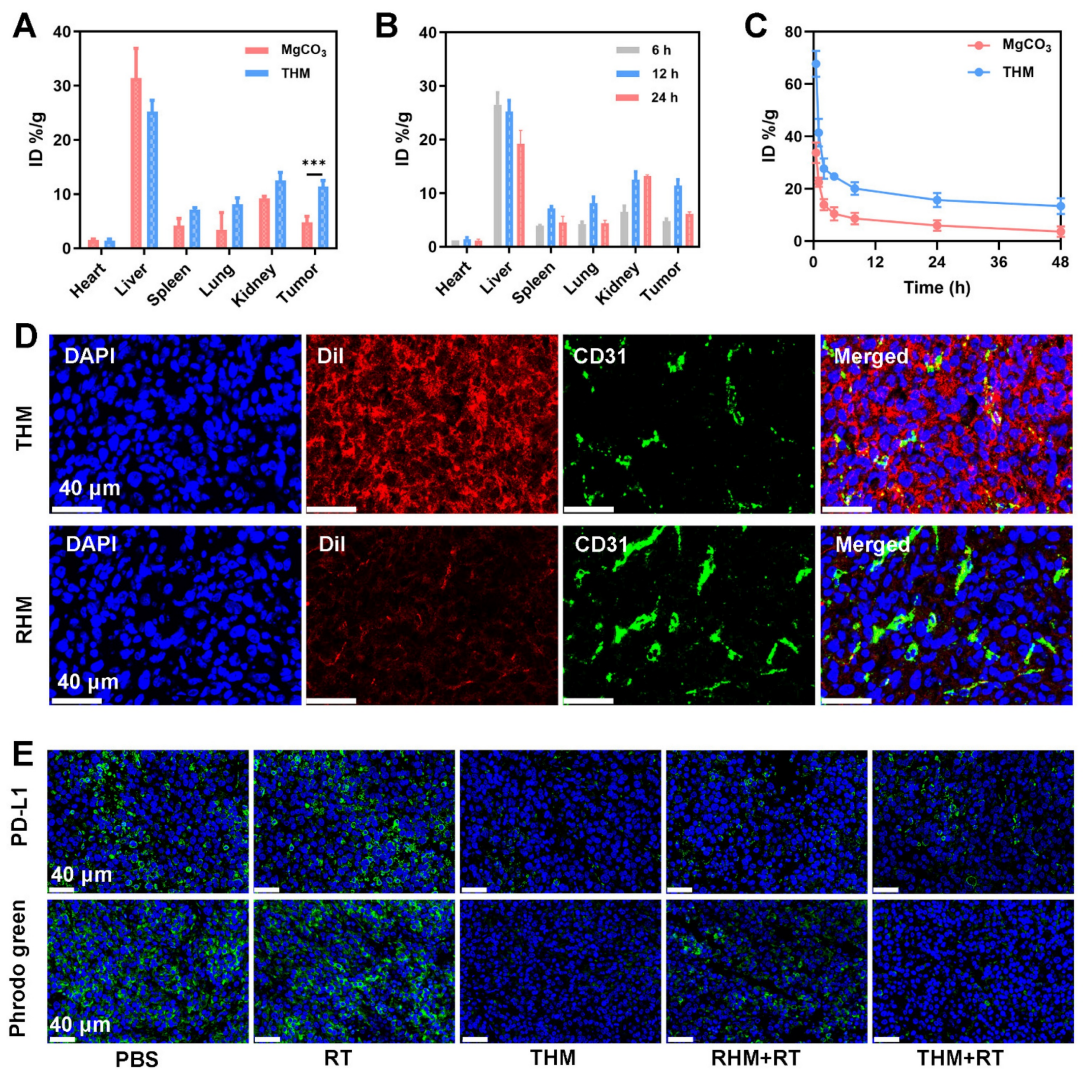
(A) *In vivo* biodistribution of indicated formulations after injections for 12h. (B) *In vivo* biodistribution of THM after injections for different times. (C) Pharmacokinetic study of MgCO_3_ nanosheets and THM over a span of 48 h. (D) Dil labeled nanoparticles and CD31 expression in tumor tissues. (E) PD-L1 expression and pHrodo green fluorescence image after different treatments. Data are shown as the mean ± SD (n = 3).

Given the promising *in vitro* anti-tumor effects of THM, we further investigated its therapeutic efficacy *in vivo*. We first examined the biodistribution of THM and its accumulation in tumor tissues. Figure 4A present Mg accumulation of tumors and major organs harvested 12 hours after injection of THM or MgCO_3_. Notably, THM group exhibited shows increased tumor accumulation, demonstrating THM's excellent targeting capability. The highest magnesium content was found at the tumor site 12 hours after THM injection (Figure 4B). The loading of T-cell membranes enabled significantly enhanced accumulation efficiency in PD-L1-high tumors. Pharmacokinetic results revealed that THM had a longer blood circulation time compared to MgCO_3_, indicating its superior immune evasion capacity (Figure 4C). The kidney is the main excretion route of magnesium 53. The half-life of blood circulation of MgCO_3_ and THM were 0.855 and 1.265 hours, respectively. Subsequent immunofluorescence analysis of tumor sections from differently treated mice showed that THM effectively penetrated tumor cells (Figure 4D), suggesting it can selectively accumulate at tumor sites through passive/active targeting mechanisms. These findings collectively demonstrate that THM serves as an ideal drug carrier for tumor-targeted delivery, offering favorable pharmacokinetic properties. Relative to the control groups, mice receiving THM and notably THM + RT exhibited markedly diminished fluorescence (Figure 4E). This observation indicates that THM effectively counteract hydrogen ions, thereby mitigating tumor acidity. Additionally, THM's binding to surface PD-L1, eliminating a critical obstacle to T cell-mediated tumor cytotoxicity. This mechanism synergistically amplifies the overall efficacy of anti-tumor immune responses.

**Figure 5:**
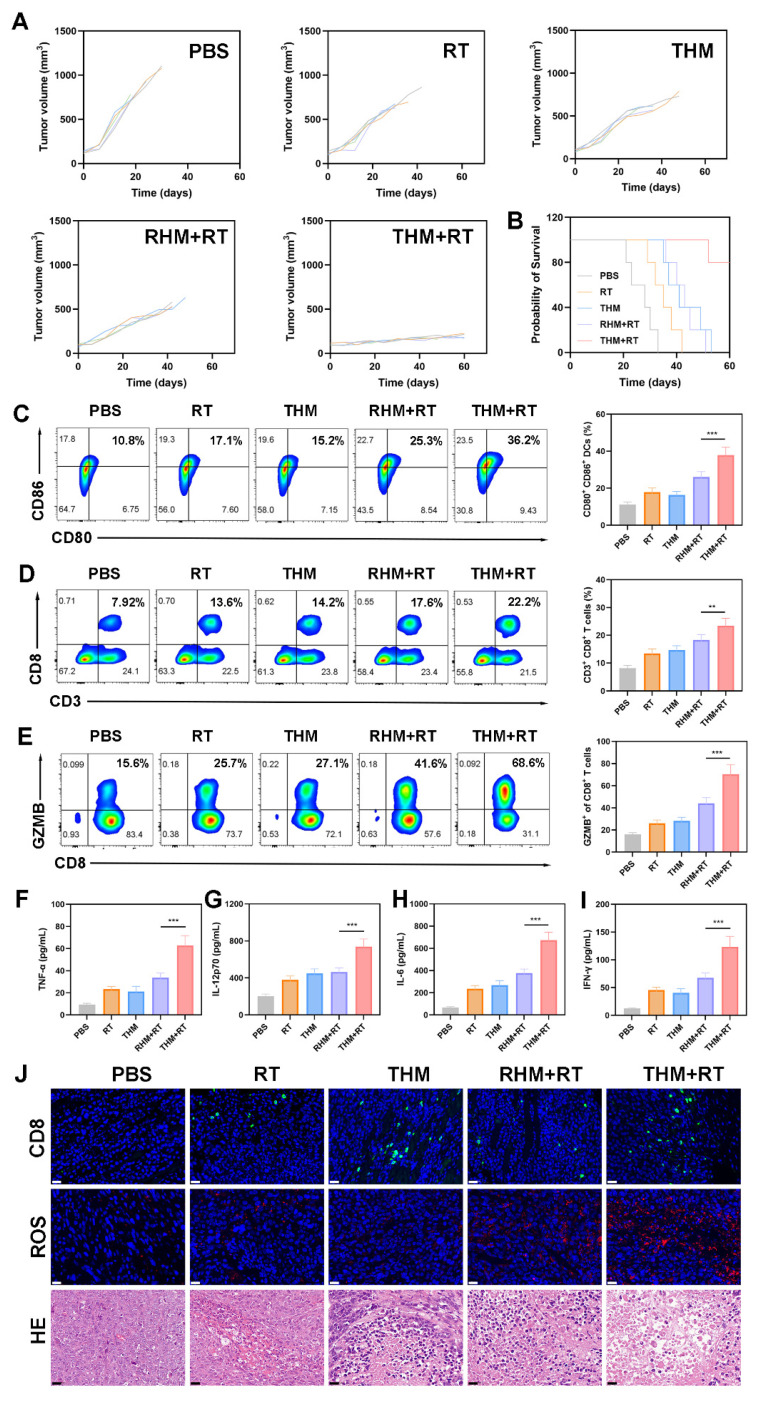
(A) Tumor volume curves and (B) survival curves in different groups. (C) Flow cytometry and quantitative analysis showing the DCs maturation in lymph nodes, (D) CD3^+^ CD8^+^ T lymphocytes in tumor tissues and (E) GZMB^+^ cells among CD8^+^ T cells. (F) Secretion of TNF-α, (G) IL-12p70, (H) IL-6 and (I) IFN-γ in sera. (J) HE, ROS and CD8^+^ staining of tumor sections. Scale bars: 40 μm. Data are shown as the mean ± SD (n = 5). ^***^p < 0.001.

Subsequently, we performed anti-tumor experiments (Figure 5A and S8). During the 60-days monitoring phase, the PBS control group displayed accelerated tumor development, whereas RT+THM exhibited intermediate tumor suppression effects (Figure S9). Although monotherapy approaches were insufficient to fully halt tumor expansion, the THM+RT achieved the most tumor inhibition, maintaining an 80% survival rate beyond 60 days (Figure 5B), thereby validating its robust *in vivo* anticancer efficacy. No histopathological alterations or biochemical deviations were identified in key organs (Figures S10 and S11), underscoring the treatment's favorable biocompatibility. To explore how THM modulates anti-tumor immunity, we analyzed DCs maturation and T-cell activation from treated mice. Flow cytometry revealed that DCs in the THM+RT group exhibited the highest expression of CD80 and CD86, significantly surpassing the PBS (10.8%), RT (17.1%), and THM (15.2%) groups (Figures 5C). Similarly, CD8^+^ T-cell proportions peaked in the THM+RT group (22.2%), representing a 2.8-fold increase over PBS (7.92%) and a 1.3-1.6-fold advantage over other treatments (Figures 5D). Notably, CD8^+^GMZB^+^ T cells surged 4.29-fold versus PBS (Figure 5E), and serum levels of inflammatory factors were markedly elevated, underscoring THM+RT's potent immunostimulatory effects (Figures 5F-5I). Immunofluorescence confirmed enhanced CD8^+^ T-cell infiltration in tumors (Figure 5J), while HE staining revealed extensive necrosis. Additionally, THM+RT correlated with heightened ROS generation and ICD induction (Figure S12), further supporting its therapeutic efficacy.

**Figure 6:**
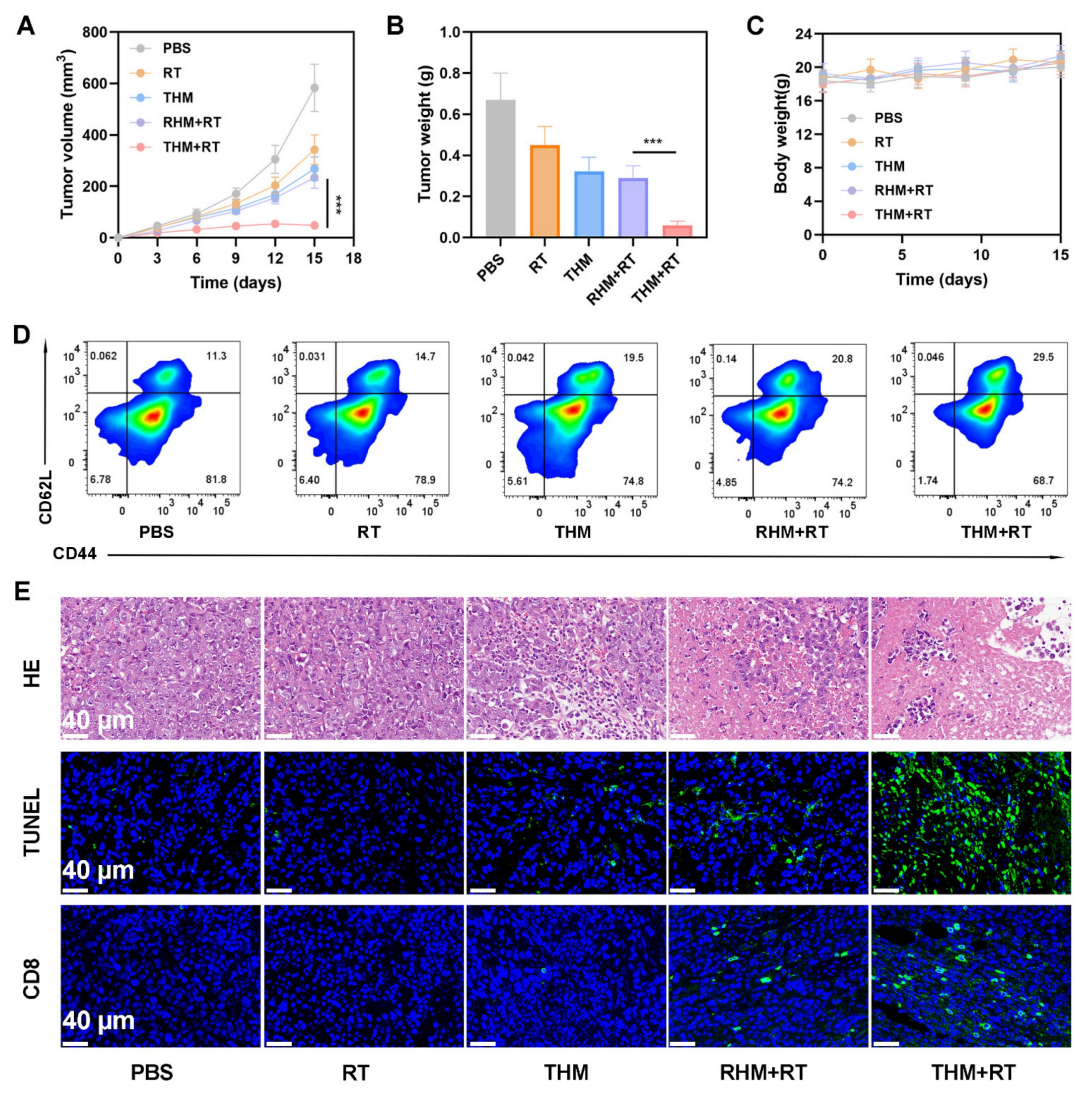
(A) Tumor volumes curve. (B) Tumor weights recorded per group at study end. (C) Body weight change (n = 5). (D) flow cytometry with quantitative analysis to determine serum central memory T cell (Tcm) proportions. (E) HE, TUNEL, and CD8⁺ staining of tumor sections. ^***^p < 0.001.

We next investigated the ability of THM to suppress tumor recurrence. In Balb/c mice, primary tumors were surgically removed following treatment with various formulations. Monitoring of recurrent tumor growth revealed that the THM+RT combination significantly delayed recurrence, whereas PBS, RT alone, or THM alone failed to prevent rapid tumor regrowth (Figures 6A and 6B). Notably, the body weight of the mice did not show a significant decrease (Figure 6C). Given that memory T cells are critical for preventing tumor recurrence by mediating long-term immune protection, we analyzed blood samples on day 14 post-treatment. THM+RT-treated mice exhibited elevated Central memory T cells (Tcm) frequencies compared to controls (Figure 6D). Histological evaluation via HE and TUNEL staining further confirmed enhanced tumor cell necrosis and apoptosis in the THM+RT group (Figure 6E). Together, these findings demonstrate that THM+RT potentiates antigen-specific anti-tumor immunity and establishes durable immunological memory to inhibit recurrence.

## Conclusions

We have engineered a bioinspired nano-system by co-loading MgCO_3_ and hemin onto T lymphocyte membranes to develop THM, a platform designed to potentiate Mg^2+^-based tumor metallo-immunotherapy. Upon intravenous delivery, THM selectively accumulates in tumors, competitively blocks PD-L1 interactions, and reverses T cell immunosuppression in the tumor microenvironment. THM persistently catalyzes H_2_O_2_ into •OH, thereby generating sustained ROS. This dual mechanism of THM, when combined with radiotherapy, induces robust ICD in tumor cells, a process that release DAMPs to drive DCs maturation and promote T cell infiltration into tumors. The alkaline MgCO_3_ component neutralizes tumor-secreted protons, effectively mitigating TME acidosis, while released Mg^2+^ enhances T cell activation and granzyme B production to facilitate the clearance of residual tumor cells. THM simultaneously remodels the tumor microenvironment, radiosensitizes tumors via ROS generation, and act as an immunomodulator to amplify the efficacy of radioimmunotherapy, thus exhibiting strong translational potential for clinical application. Future investigations will focus on exploring THM's application as a vaccine adjuvant for tumor antigen delivery and its utility in prophylactic cancer therapy.

## Supplementary Material

Supplementary figures.

## Figures and Tables

**Scheme 1:**
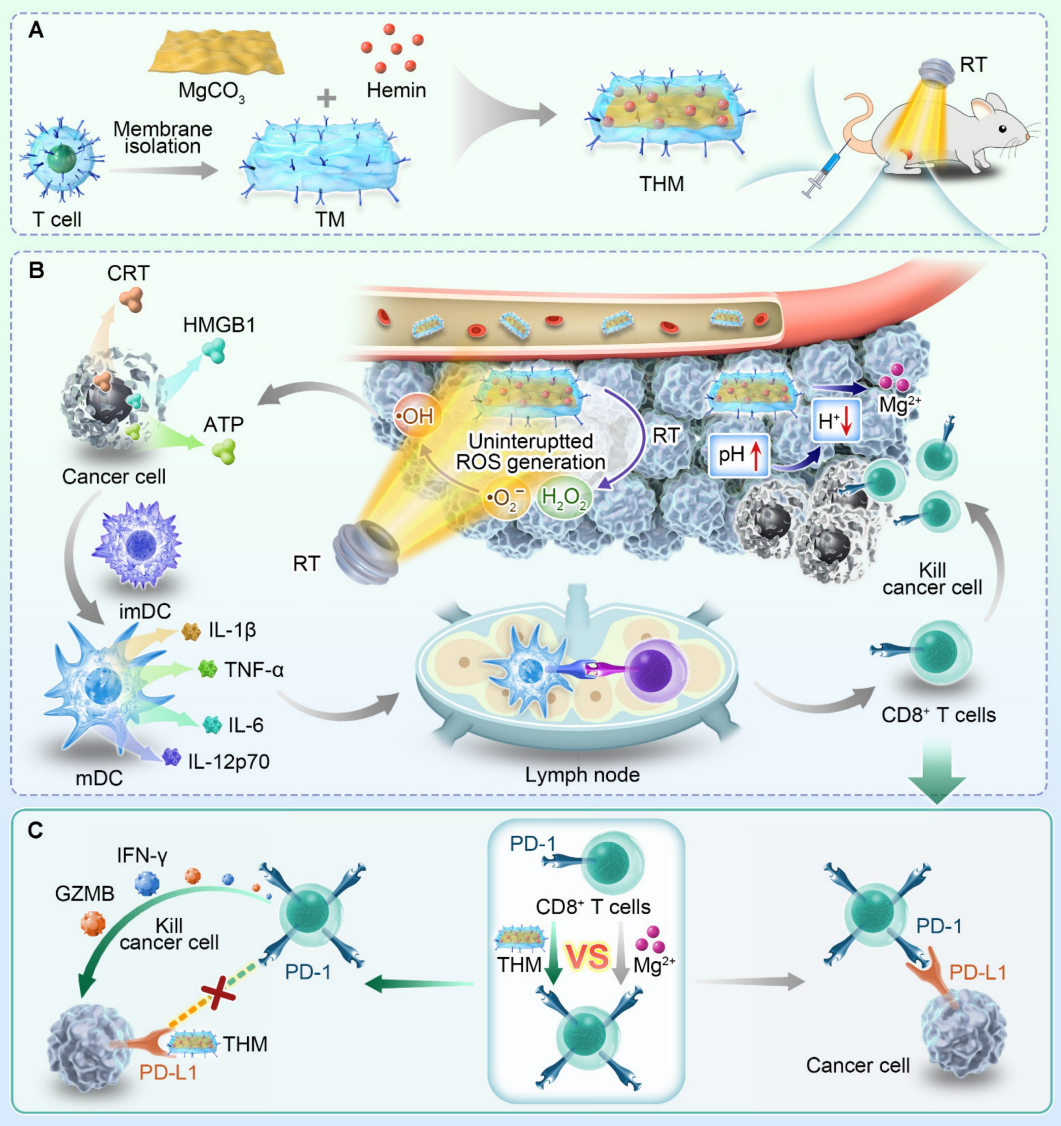
Schematic illustration of T lymphocyte membrane bionic nanomedicine synergizes with radiotherapy for enhancing Mg^2+^-mediated tumor metallo-immunotherapy and preventing recurrence. (A) Schematic diagram of the preparation process of THM and treatment in mice. (B) The *in vivo* anti-tumor mechanism mediated by THM. (C) Schematic diagram of THM activating T lymphocytes.

## Abbreviations

TCM: T cell membranes
Tcm: Central memory T cells
RT: Radiotherapy
ROS: Reactive oxygen species
HMGB1: High mobility group protein B1
CRT: Calreticulin
DAMPs: Damage Associated Molecular Patterns
GZMB: Granzyme B
BMDCs: Bone Marrow-Derived Dendritic Cells
CLSM: Confocal Laser Scanning Microscopy
