# Supplementary material for: T lymphocyte membrane bionic nanomedicine synergizes with radiotherapy for enhancing Mg2+-mediated tumor metallo-immunotherapy and preventing recurrence
Source: Theranostics. 2026 Jan 8;16(7):3634–47. doi: 10.7150/thno.127717 (PMC12846755; doi:10.7150/thno.127717)
Supplement: Supplementary file 1 — Supplementary figures. [file thnov16p3634s1.pdf]

## Supplementary figures

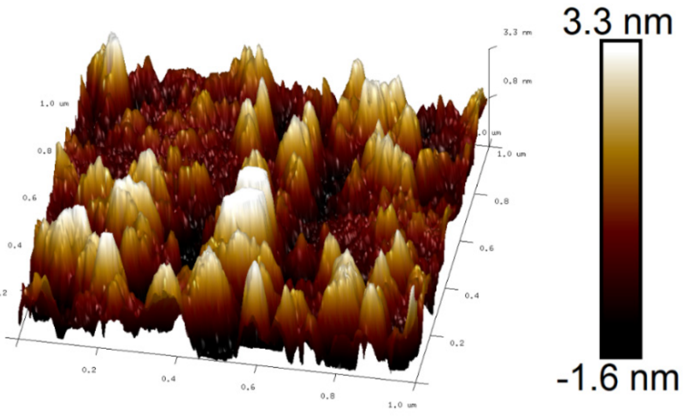


**Figure S1.** AFM image of the $MgCO_3$ nanosheet and height profiles along the dashed lines in AFM image.

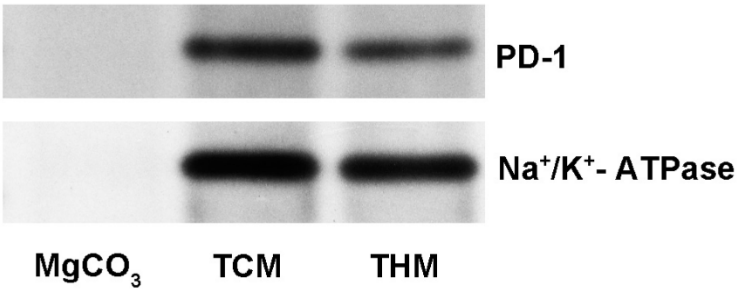


**Figure S2.** Western blot analysis of $Na^+/K^+$- ATPase and PD-1 proteins in different formulations.

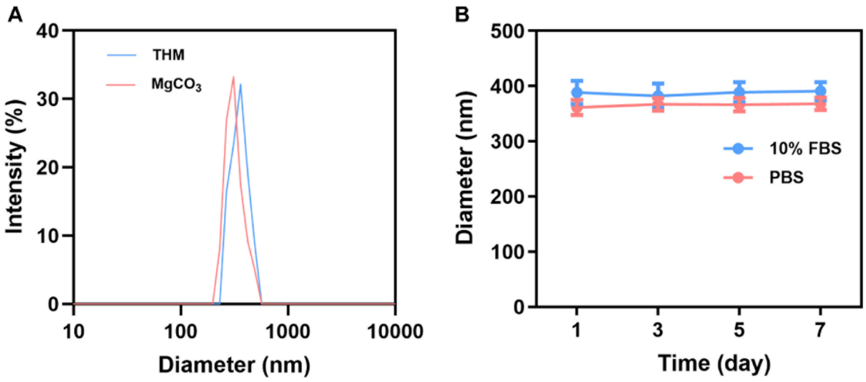


**Figure S3.** (A) The particle size distribution of different nanomaterials. (B) Stability of

THM in various solutions at different times. Data are shown as the mean ± SD (n = 3).

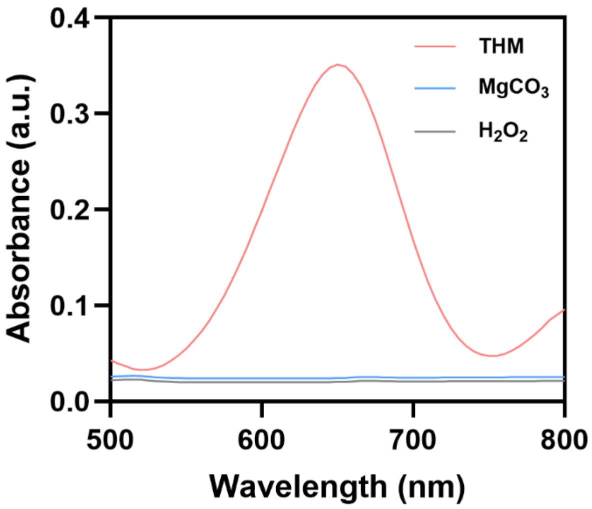


**Figure S4.** Oxidation of TMB due to generated •OH by indicated treatments.

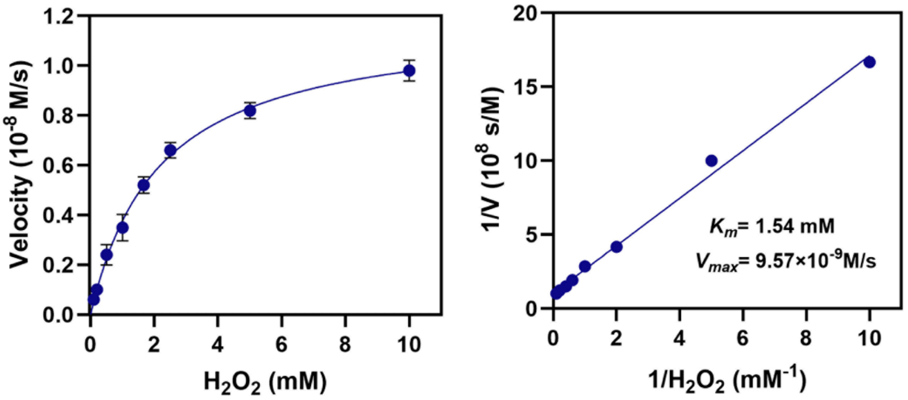


**Figure S5.** Initial velocity of the reaction with varied concentration of $H_2O_2$ and the double-reciprocal plots of $H_2O_2$. Data are shown as the mean ± SD (n = 3)**.**

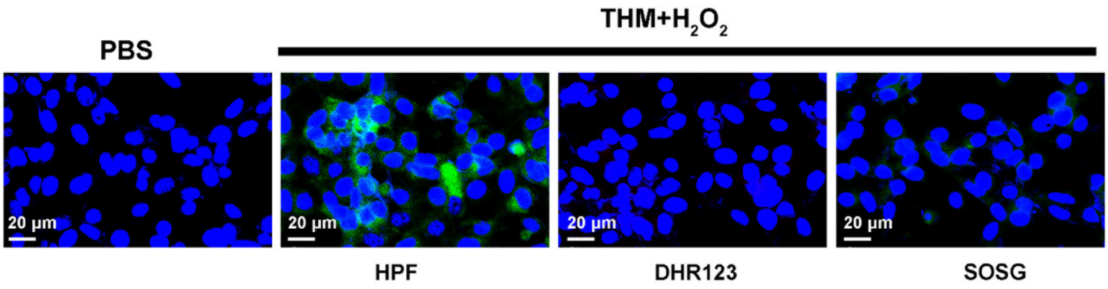


**Figure S6.** CLSM images of different types of ROS generated in 4T1 cells upon

different treatments. Blue: DAPI.

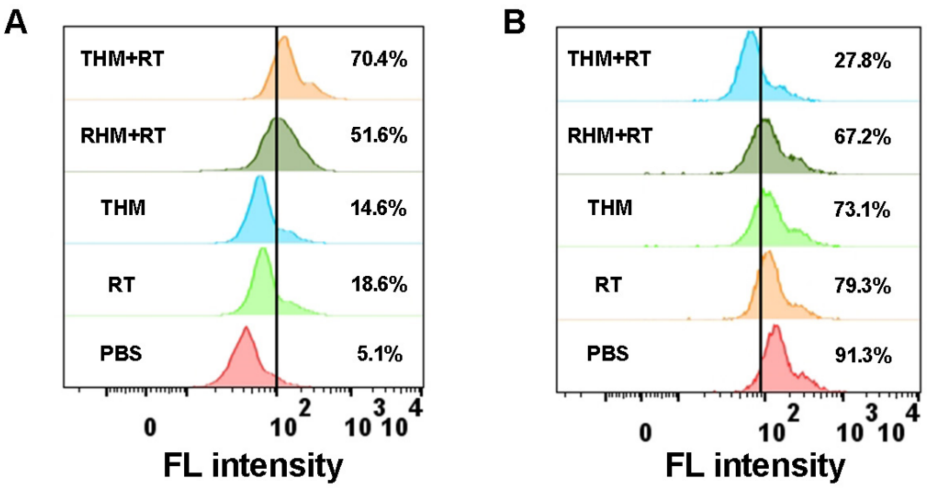


**Figure S7.** (A) Fluorescence-activated cell sorting (FACS) micrograph of CRT and (B) HMGB1 expression in nanoparticles-treated 4T1 cells.

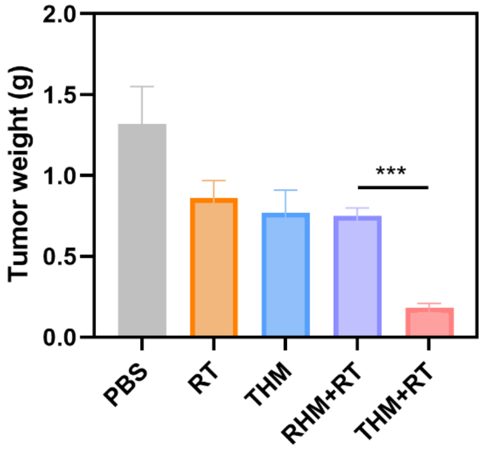


**Figure S8.** Tumor weight after different treatments. Data are shown as the mean ± SD (n = 5).

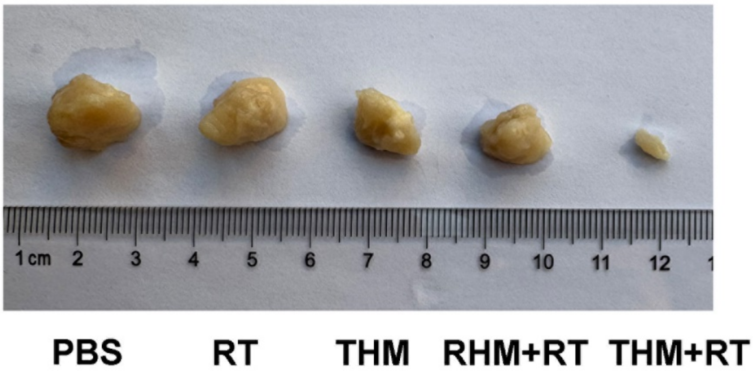

**Figure S9.** Representative tumor pictures after different treatments.

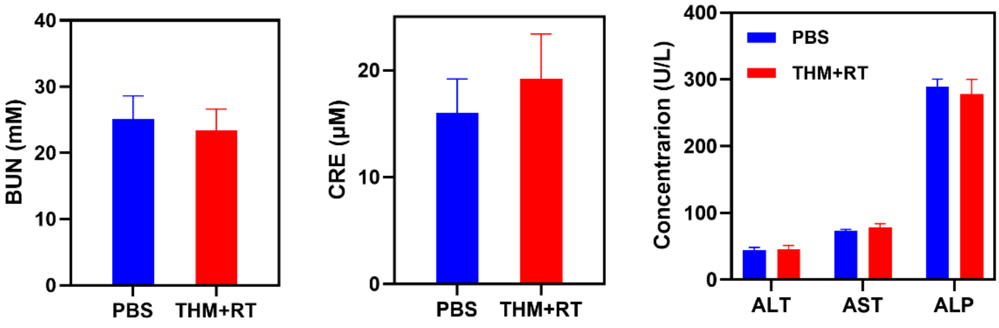


**Figure S10.** Analysis of liver and kidney function indicators. Data are shown as the mean ± SD (n = 5).

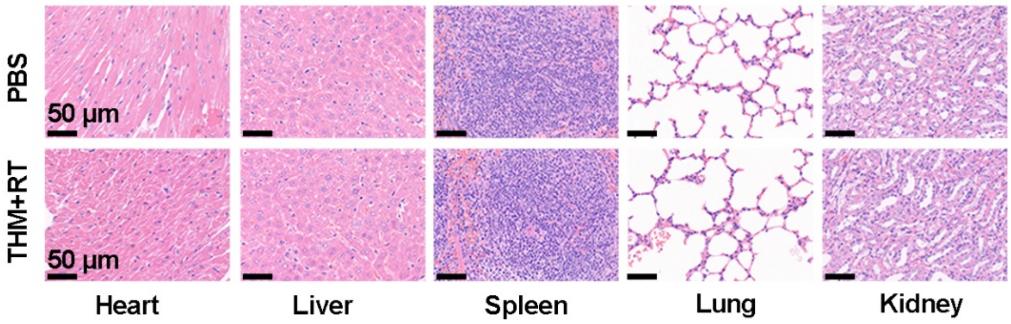


**Figure S11.** HE sections of major organs after different treatments.

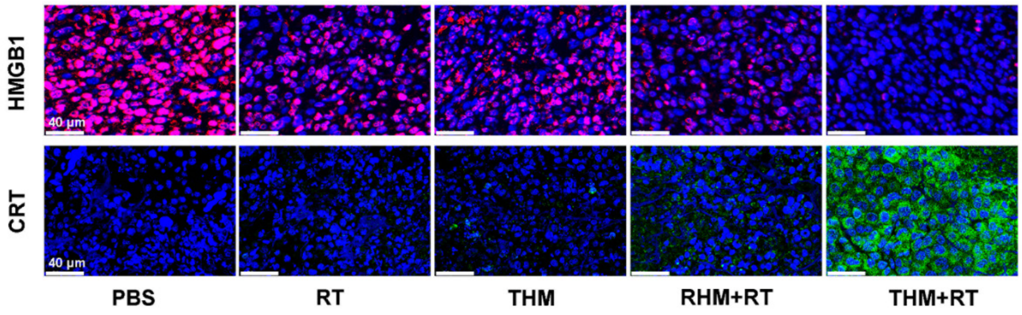


**Figure S12.** CRT and HMGB1 staining of tumor sections after the indicated treatments.
